# 2-Chloro­pyridine-3-carboxamide

**DOI:** 10.1107/S1600536809002256

**Published:** 2009-01-23

**Authors:** Yu-Peng Hua, Ying Xu, Xue-Hong Wei, Hong-Bo Tong

**Affiliations:** a Institute of Applied Chemistry, Shanxi University, Taiyuan 030006, People’s Republic of China

## Abstract

In the crystal structure of the title compound, C_6_H_5_ClN_2_O, the dihedral angle between the pyridine ring and the carboxamine group is 63.88 (8)°. Inter­molecular N—H⋯N and N—H⋯O hydrogen bonds link the mol­ecules into a two-dimensional network.

## Related literature

Details of applications of the title compound can be found in: Oda *et al.* (1993 ▶); Qin *et al.* (2001 ▶).
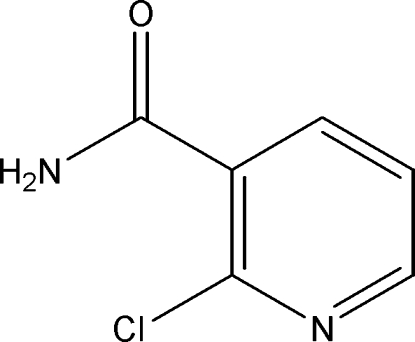


## Experimental

### 

#### Crystal data

C_6_H_5_ClN_2_O
*M* *_r_* = 156.57
Monoclinic, 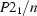

*a* = 6.980 (5) Å
*b* = 13.627 (9) Å
*c* = 7.108 (5) Å
β = 91.82 (5)°
*V* = 675.8 (8) Å^3^
*Z* = 4
Mo *K*α radiation
μ = 0.49 mm^−1^
*T* = 293 (2) K
0.30 × 0.20 × 0.20 mm

#### Data collection

Siemens SMART CCD area-detector diffractometer
Absorption correction: multi-scan (*SADABS*; Sheldrick, 1997 ▶) *T*_min_ = 0.868, *T*_max_ = 0.909
2716 measured reflections
1188 independent reflections
1083 reflections with *I* > 2σ(*I*)
*R*_int_ = 0.021

#### Refinement

*R*[*F* ^2^ > 2σ(*F* ^2^)] = 0.034
*wR*(*F* ^2^) = 0.096
*S* = 1.11
1188 reflections
92 parameters
H-atom parameters constrained
Δρ_max_ = 0.18 e Å^−3^
Δρ_min_ = −0.23 e Å^−3^

### 

Data collection: *SMART* (Siemens, 1996 ▶); cell refinement: *SAINT* (Siemens, 1996 ▶); data reduction: *SAINT*; program(s) used to solve structure: *SHELXS97* (Sheldrick, 2008 ▶); program(s) used to refine structure: *SHELXL97* (Sheldrick, 2008 ▶); molecular graphics: *SHELXTL* (Sheldrick, 2008 ▶); software used to prepare material for publication: *SHELXL97*.

## Supplementary Material

Crystal structure: contains datablocks I, global. DOI: 10.1107/S1600536809002256/nc2131sup1.cif

Structure factors: contains datablocks I. DOI: 10.1107/S1600536809002256/nc2131Isup2.hkl

Additional supplementary materials:  crystallographic information; 3D view; checkCIF report

## Figures and Tables

**Table 1:** Hydrogen-bond geometry (Å, °)

*D*—H⋯*A*	*D*—H	H⋯*A*	*D*⋯*A*	*D*—H⋯*A*
N2—H2*A*⋯N1^i^	0.86	2.21	3.003 (3)	154
N2—H2*B*⋯O^ii^	0.86	2.17	3.015 (3)	168

Symmetry codes: (i) 
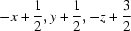
; (ii) 
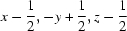
.

**Fig. 1.**
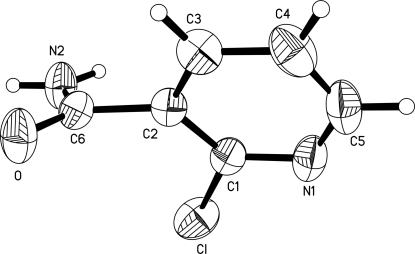
View of the molecule of the title compound showing the atom-labelling scheme. Displacement ellipsoids are drawn at the 50% probability level.

**Fig. 2.**
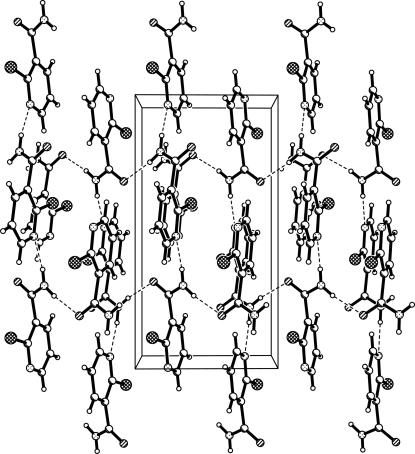
Crystal structure of the title compound along [100] with intermolecular N—H···N and N—H···O hydrogen bonding shown as dashed lines.

## supplementary crystallographic information

### Comment

The structure of 2-chloropyridine-3-carboxamide has attracted us owing to its fungicidal activities (Oda *et al.*, 1993) and its application in coordination chemistry (Qin *et al.*, 2001). The dihedral angles formed by the pyridine ring and the carboxamine group amount to 63.88 (8)° (Fig. 1). The molecules are connected via intermolecular N—H···N and N—H···O hydrogen bonding into layers, with H···N distances of 2.21 and O···H distances of 2.17 Å (Fig. 2 and Tab. 1).

### Experimental

Ammonia (10 ml, 66 mmol, 25%) was added slowly to a solution of 2-chloropyridine-3-carbonyl chloride (4.0 g, 22 mmol) in THF (20 ml) at 0°C. The reaction mixture was allowed to warm up to room temperature and stirred for 1.5 h. The resulting mixture was dried under vacuum and washed with two 20 ml portions of THF. Then the solution was dried over anhydrous magnesium sulfate. The solvent was removed by vacuum, and the product was collected, yield: 1.93 g, 56%; m.p. 162.5°C. The crystal suitable for X-ray analysis was grown by slow evaporation of the solvent from a diethyl ether solution at 20°. Anal. Calcd for C_6_H_5_ClN_2_O: C, 45.97; H, 3.14; N, 17.82%. Found: C, 46.03; H, 3.22; N, 17.89%.

### Refinement

All H atoms were positioned with idealized geometry, with C—H = 0.96 and N—H = 0.86 Å, and were refined with *U*_iso_(H) values set to 1.2 U_eq_(C,N).

### Crystal data

**Table:**

C_6_H_5_ClN_2_O	*F*(000) = 320
*M**_r_* = 156.57	*D*_x_ = 1.539 Mg m^−^^3^
Monoclinic, *P*2_1_/*n*	Mo *K*α radiation, λ = 0.71073 Å
*a* = 6.980 (5) Å	Cell parameters from 1764 reflections
*b* = 13.627 (9) Å	θ = 2.9–26.9°
*c* = 7.108 (5) Å	µ = 0.49 mm^−^^1^
β = 91.82 (5)°	*T* = 293 K
*V* = 675.8 (8) Å^3^	Plate, yellow
*Z* = 4	0.30 × 0.20 × 0.20 mm

### Data collection

**Table:**

Siemens SMART CCD area-detector diffractometer	1188 independent reflections
Radiation source: fine-focus sealed tube	1083 reflections with *I* > 2σ(*I*)
graphite	*R*_int_ = 0.021
ω scans	θ_max_ = 25.0°, θ_min_ = 3.0°
Absorption correction: multi-scan (*SADABS*; Sheldrick, 1997)	*h* = −8→6
*T*_min_ = 0.868, *T*_max_ = 0.909	*k* = −15→16
2716 measured reflections	*l* = −6→8

### Refinement

**Table:**

Refinement on *F*^2^	Secondary atom site location: difference Fourier map
Least-squares matrix: full	Hydrogen site location: inferred from neighbouring sites
*R*[*F*^2^ > 2σ(*F*^2^)] = 0.034	H-atom parameters constrained
*wR*(*F*^2^) = 0.096	*w* = 1/[σ^2^(*F*_o_^2^) + (0.0551*P*)^2^ + 0.1035*P*] where *P* = (*F*_o_^2^ + 2*F*_c_^2^)/3
*S* = 1.11	(Δ/σ)_max_ < 0.001
1188 reflections	Δρ_max_ = 0.18 e Å^−^^3^
92 parameters	Δρ_min_ = −0.23 e Å^−^^3^
0 restraints	Extinction correction: *SHELXL97* (Sheldrick, 2008), Fc^*^=kFc[1+0.001xFc^2^λ^3^/sin(2θ)]^-1/4^
Primary atom site location: structure-invariant direct methods	Extinction coefficient: 0.051 (8)

### Special details

**Table:**

Geometry. All e.s.d.'s (except the e.s.d. in the dihedral angle between two l.s. planes) are estimated using the full covariance matrix. The cell e.s.d.'s are taken into account individually in the estimation of e.s.d.'s in distances, angles and torsion angles; correlations between e.s.d.'s in cell parameters are only used when they are defined by crystal symmetry. An approximate (isotropic) treatment of cell e.s.d.'s is used for estimating e.s.d.'s involving l.s. planes.
Refinement. Refinement of *F*^2^ against ALL reflections. The weighted *R*-factor *wR* and goodness of fit *S* are based on *F*^2^, conventional *R*-factors *R* are based on *F*, with *F* set to zero for negative *F*^2^. The threshold expression of *F*^2^ > σ(*F*^2^) is used only for calculating *R*-factors(gt) *etc*. and is not relevant to the choice of reflections for refinement. *R*-factors based on *F*^2^ are statistically about twice as large as those based on *F*, and *R*- factors based on ALL data will be even larger.

### Fractional atomic coordinates and isotropic or equivalent isotropic displacement parameters (Å^2^)

**Table:**

	*x*	*y*	*z*	*U*_iso_*/*U*_eq_	
Cl	0.14993 (7)	0.10791 (4)	0.88571 (7)	0.0533 (2)	
C1	0.3716 (2)	0.07827 (13)	0.7965 (2)	0.0349 (4)	
N1	0.4064 (2)	−0.01655 (11)	0.7824 (2)	0.0446 (4)	
O	0.5546 (2)	0.30761 (9)	0.8932 (2)	0.0507 (4)	
C6	0.4574 (2)	0.25974 (12)	0.7786 (2)	0.0351 (4)	
N2	0.3180 (2)	0.29749 (11)	0.6708 (2)	0.0446 (4)	
H2A	0.2909	0.3589	0.6786	0.054*	
H2B	0.2545	0.2606	0.5930	0.054*	
C3	0.6760 (3)	0.12278 (14)	0.6910 (3)	0.0430 (5)	
H3	0.7681	0.1692	0.6616	0.052*	
C4	0.7153 (3)	0.02376 (16)	0.6735 (3)	0.0505 (5)	
H4	0.8331	0.0026	0.6312	0.061*	
C2	0.4989 (2)	0.15240 (12)	0.7524 (2)	0.0325 (4)	
C5	0.5774 (3)	−0.04231 (14)	0.7198 (3)	0.0506 (6)	
H5	0.6042	−0.1088	0.7071	0.061*	

### Atomic displacement parameters (Å^2^)

**Table:**

	*U*^11^	*U*^22^	*U*^33^	*U*^12^	*U*^13^	*U*^23^
Cl	0.0357 (3)	0.0599 (4)	0.0647 (4)	−0.0034 (2)	0.0066 (2)	0.0116 (2)
C1	0.0347 (9)	0.0346 (9)	0.0350 (9)	−0.0023 (7)	−0.0060 (7)	0.0017 (7)
N1	0.0541 (10)	0.0302 (8)	0.0485 (9)	−0.0029 (7)	−0.0125 (8)	0.0009 (6)
O	0.0548 (9)	0.0356 (7)	0.0603 (9)	−0.0041 (6)	−0.0181 (7)	−0.0060 (6)
C6	0.0333 (9)	0.0319 (9)	0.0399 (9)	−0.0025 (7)	−0.0003 (7)	0.0020 (7)
N2	0.0456 (9)	0.0300 (8)	0.0573 (10)	0.0039 (6)	−0.0124 (8)	−0.0020 (7)
C3	0.0336 (10)	0.0493 (12)	0.0460 (10)	0.0009 (8)	−0.0022 (8)	−0.0017 (8)
C4	0.0431 (11)	0.0566 (13)	0.0512 (12)	0.0162 (9)	−0.0069 (9)	−0.0109 (9)
C2	0.0298 (9)	0.0335 (9)	0.0337 (9)	0.0002 (7)	−0.0051 (7)	−0.0002 (7)
C5	0.0649 (14)	0.0346 (10)	0.0509 (11)	0.0130 (9)	−0.0191 (10)	−0.0081 (8)

### Geometric parameters (Å, °)

**Table:**

Cl—C1	1.738 (2)	N2—H2B	0.8600
C1—N1	1.319 (2)	C3—C4	1.383 (3)
C1—C2	1.388 (3)	C3—C2	1.385 (3)
N1—C5	1.334 (3)	C3—H3	0.9300
O—C6	1.230 (2)	C4—C5	1.366 (3)
C6—N2	1.324 (2)	C4—H4	0.9300
C6—C2	1.504 (3)	C5—H5	0.9300
N2—H2A	0.8600		
			
N1—C1—C2	125.08 (18)	C4—C3—H3	120.2
N1—C1—Cl	115.07 (14)	C2—C3—H3	120.2
C2—C1—Cl	119.80 (14)	C5—C4—C3	118.6 (2)
C1—N1—C5	116.88 (16)	C5—C4—H4	120.7
O—C6—N2	123.86 (17)	C3—C4—H4	120.7
O—C6—C2	119.55 (15)	C3—C2—C1	116.34 (17)
N2—C6—C2	116.57 (15)	C3—C2—C6	120.00 (16)
C6—N2—H2A	120.0	C1—C2—C6	123.56 (16)
C6—N2—H2B	120.0	N1—C5—C4	123.49 (18)
H2A—N2—H2B	120.0	N1—C5—H5	118.3
C4—C3—C2	119.63 (19)	C4—C5—H5	118.3

### Hydrogen-bond geometry (Å, °)

**Table:**

*D*—H···*A*	*D*—H	H···*A*	*D*···*A*	*D*—H···*A*
N2—H2A···N1^i^	0.86	2.21	3.003 (3)	154
N2—H2B···O^ii^	0.86	2.17	3.015 (3)	168

Symmetry codes: (i) −*x*+1/2, *y*+1/2, −*z*+3/2; (ii) *x*−1/2, −*y*+1/2, *z*−1/2.
